# Efficacy and safety of rivaroxaban plus aspirin compared with aspirin alone in patients with peripheral arterial disease: a systematic review and meta-analysis

**DOI:** 10.1097/MS9.0000000000005050

**Published:** 2026-06-11

**Authors:** Muhammad Farhan, Syed Hassan Ali, Daniyal Khalid, Adina Kazmi, Dua Asim, Zoha Idrees, Muhammad Saim Siddiqui, Sudais Bin Sohaib, Muhammad Saad Khan, Muhammad Mohsin Khan, Abubakr Mahmoud

**Affiliations:** a Department of Medicine, Dow University of Health and Sciences, Karachi, Pakistan; b Department of Medicine, Liaquat University of Medical and Health Sciences, Hyderabad, Pakistan; c Department of Medicine, Jinnah Sindh Medical University, Karachi, Pakistan; d Department of Medicine, University of Khartoum, Khartoum State, Sudan

**Keywords:** aspirin, dual pathway inhibition, meta-analysis, peripheral artery disease, rivaroxaban

## Abstract

**Background:**

Peripheral artery disease (PAD) is a prevalent atherosclerotic disorder associated with significant cardiovascular morbidity. Although aspirin remains the cornerstone of therapy, residual thrombotic risk persists. The addition of rivaroxaban, a factor Xa inhibitor, may enhance protection through dual pathway inhibition. This meta-analysis aimed to evaluate the efficacy and safety of rivaroxaban plus aspirin versus aspirin alone in patients with PAD.

**Methods:**

A systematic review and meta-analysis were conducted following PRISMA and AMSTAR guidelines. Randomized controlled trials comparing rivaroxaban plus aspirin with aspirin alone in adults with PAD were included. Data were pooled using a random-effects model in Review Manager 5.4. Risk of bias was assessed with RoB 2 and certainty of evidence was graded using GRADE pro.

**Results:**

Six RCTs including 90 325 participants met inclusion criteria. Combination therapy significantly reduced MACE (RR: 0.82, 95% CI: 0.78–0.87), MI (RR: 0.87), ischemic stroke (RR: 0.76), and acute limb ischemia (RR: 0.68), with low heterogeneity (≤35%). No significant reduction was observed in cardiovascular mortality or major amputation. TIMI major bleeding risk increased (RR: 1.41, 95% CI: 1.07–1.84), however intracranial and fatal bleeding rates were similar between groups. Moderate-to-high certainty evidence supports reductions in ischemic and limb events, with increased bleeding risk.

**Conclusion:**

Rivaroxaban plus aspirin is associated with improved cardiovascular and limb outcomes compared to aspirin alone in PAD, albeit with a higher risk of major bleeding. Findings should be interpreted in the context of potential publication bias and heterogeneity across trials. Careful patient selection is warranted for optimal risk-benefit balance.

## Introduction

Peripheral artery disease (PAD) is defined by partial or total blockage of at least one peripheral artery. It is also referred to as peripheral vascular disease, peripheral arterial occlusive disease, and lower extremity arterial disease^[^1^]^. PAD comprises a spectrum of disorders characterized by the progressive narrowing, obstruction, or aneurysmal enlargement of the aorta and its noncardiac branches. This includes the carotid branches of the upper extremities, vessels supplying the lower limb, and the arteries of visceral organs^[^2^]^. PAD is a progressive atherosclerotic disorder; it can be asymptomatic but significantly leads to functional impairment, limb-threatening ischemia, or even amputation^[^3^]^.

HIGHLIGHTS

- Rivaroxaban plus aspirin significantly reduced major adverse cardiovascular events (RR = 0.82) in patients with peripheral artery disease.
- Combination therapy lowered risks of myocardial infarction (RR = 0.87), ischemic stroke (RR = 0.76), and acute limb ischemia (RR = 0.68).
- No significant difference was observed in cardiovascular mortality or major amputation rates.
- TIMI major bleeding risk increased by 41%, however fatal and intracranial bleeding remained comparable between groups.
- Dual pathway inhibition offers superior vascular protection but requires individualized risk–benefit assessment.

PAD has emerged as a significant global health concern in the twenty-first century, with older adults bearing the greatest burden^[^4^]^. According to the Global Burden of Disease Study 2019, an estimated 113 million individuals aged 40 years and above lived with PAD, reflecting a global prevalence of 1.52%^[^4,5^]^. Approximately 20% of adults aged ≥80 years are affected, the condition is rarely detected before 60 years of age; however, prevalence increases substantially with advancing age, peaking between 70 and 74 years^[^4,6^]^. After ischemic heart disease and stroke, PAD is the third leading cause of atherosclerotic morbidity globally^[^7^]^. The prevalence of PAD rose from 65.76 million to 113.44 million cases between 1990 and 2019, a 72% rise. During this period, crude prevalence per 100 000 increased by 13%, whereas the age-standardized prevalence declined by 22%, reflecting a lower age-adjusted risk^[^8^]^. PAD prevalence demonstrated a positive association with advancing age; at ages 40–44 years, it was slightly higher in low-income and middle-income countries (4.32%) than in high-income countries (3.54%). However, among individuals aged 80–84 years, prevalence was notably higher in high-income countries (21.24% vs 12.04%). In high-income countries, women have a slightly higher prevalence than men up to 75 years of age do; in low-income and middle-income countries, sex differences are minimal. Overall, global prevalence among those aged 25 years and older is 5.56%, higher in high-income (7.37%) than low-income and middle-income countries (5.09%)^[^9^]^.

Approximately 70% of PAD cases are attributable to conventional atherogenic risk factors including advancing age, hypertension, dyslipidemia, cigarette smoking, and diabetes. Evidence indicates that a 1% increase in hemoglobin A1c is associated with a 26% increase in the risk of developing PAD. Moreover, individuals without diabetes may also exhibit elevated susceptibility to PAD in the context of insulin resistance^[^10^]^. Major adverse cardiovascular (CV) events (MACE), such as myocardial infarction (MI), stroke, and CV death, are more frequently observed in patients with PAD. In addition to MACE, patients with PAD also suffer from extremity dysfunctions and complications, which serve as a primary cause of morbidity. Reduced perfusion in the lower extremities often leads to claudication and impaired walking performance. Severe impairment can lead to critical limb ischemia and increase the risk of major adverse limb events such as peripheral revascularization, amputation, and acute limb ischemia (ALI)^[^11^]^.

The severity of PAD is intimately linked to elevated vascular reactivity and enhanced coagulation system activity, both of which are frequently seen in patients with PAD^[^12^]^. Platelet hyperactivity has been frequently observed, accompanied by alterations in fibrinogen levels, thrombin generation, and fibrin turnover. In patients with critical limb-threatening ischemia, levels of natural anticoagulants such as protein C and protein S are often reduced. In addition, concentrations of coagulation factors IX, XI, and XII are also commonly decreased^[^13^]^.

Since PAD is a serious condition that has lifelong implications for patients, imaging modalities are crucial for early diagnosis, effective treatment, and recurrence prevention. Common imaging techniques used in PAD diagnosis include DUS, MRA, DSA, and CTA and DSA. On imaging, the arterial disease presents with a lipid rich necrotic core (LRNC), intraplaque hemorrhage (IPH), calcification, intermittent claudication, chronic limb threatening ischemia (CLTI),rest pain, foot ulcers, or ischemic gangrene^[^14^]^. Additionally, the ankle brachial index (ABI), a simple test that measures the blood pressure in your arm and ankle, is widely used by doctors. If the ABI value is below 0.9, it usually means there’s a higher chance of heart related problems or even death, sometimes up to four times more likely^[^15^]^.

Lifestyle changes such as quitting smoking and eating healthy play a key role in managing PAD. Medications that lower cholesterol, prevent clots, or manage blood sugar in diabetic patients are all key tools used to cut down the risks that come with PAD^[^11^]^. Endovascular treatments and, in cases of extensive arterial involvement, surgical operations are the first line therapeutic options for PAD. Endovascular treatments such as balloon angioplasty, drug coated balloons, and drug coated stents serve as minimally invasive techniques, whereas surgical procedures, though more complex, remain the gold standard for long and intricate lesions. However, beyond these interventions, medical management plays a key role in reducing CV risk in PAD patients^[^14^]^.

Aspirin is frequently used to help avoid major CV problems since it can lower the formation of blood clots. Clinical studies have shown that aspirin can lower the risk of major heart problems by around 19%, and the risk of death from heart disease by around 9% compared to those not taking it. Rivaroxaban, which prevents venous clots and lowers the risk of stroke in individuals with irregular heartbeats, acts by blocking factor Xa, a crucial component of blood coagulation^[^16^]^. In the COMPASS trial, patients with PAD or heart disease who added low-dose rivaroxaban (twice daily) to their standard aspirin therapy had better outcomes, with a greater reduction in heart-related deaths, heart attacks, and strokes than those who took aspirin alone^[^17^]^.

A randomized, placebo-controlled, double-blind clinical trial with a 2 × 2 factorial design in 366 outpatients with stage I–II PAD showed that aspirin (100 mg daily) significantly reduced major vascular events (7/185 vs 20/181; risk reduction 64%, *P* = 0.022) and critical leg ischemia (12 vs 28; *P* = 0.014), with no significant increase in adverse events. In contrast, antioxidant vitamins showed no benefit (16/185 vs 11/181 vascular events), and their use was not associated with improved outcomes. Inclusion of this trial in a meta-analysis rendered the overall results highly significant (*P* < 0.001) and suggests that low-dose aspirin reduces vascular events by 26% and should routinely be considered for PAD patients, including those with type 2 diabetes^[^18^]^.

To further evaluate antithrombotic strategies in PAD, a double-blind trial randomized 6564 post-revascularization patients to receive rivaroxaban (2.5 mg twice daily) plus aspirin or placebo plus aspirin. The combination of rivaroxaban and aspirin resulted in a statistically significant reduction in the composite outcome of ALI, major vascular amputation, MI, ischemic stroke, or CV death (hazard ratio: 0.85; 95% CI: 0.76–0.96; *P* = 0.009). The incidence of the principal safety outcome of TIMI major bleeding was not significantly higher (hazard ratio: 1.43; 95% CI: 0.97 to 2.10; *P* = 0.07), but rivaroxaban plus aspirin was linked to an increased risk of ISTH major bleeding as a secondary safety outcome (hazard ratio: 1.42; 95% CI: 1.10 to 1.84; *P* = 0.007)^[^19^]^.

Further supporting the benefit of combination therapy, a subanalysis from the Cardiovascular Outcomes for People Using Anticoagulation Strategies (COMPASS) trial showed that in patients with symptomatic lower-extremity PAD (LE-PAD), rivaroxaban plus aspirin reduced MACE or major adverse limb events by 4.2% compared to aspirin alone. The estimated absolute risk increase of major bleeding was 2.0%, and for fatal or critical organ bleeding was 0.4%, such that the net clinical benefit was estimated to be 3.2%^[^20^]^.

Despite robust evidence from large randomized trials such as COMPASS and VOYAGER-PAD demonstrating the benefit of dual pathway inhibition in patients with PAD, uncertainty regarding the consistency of benefit across different CV and limb-related outcomes and the balance between ischemic protection and bleeding risk remains. Furthermore, existing reviews often emphasize individual trials or selected endpoints, limiting a comprehensive synthesis of randomized evidence focused specifically on PAD populations. Therefore, this systematic review and meta-analysis study was undertaken to pool randomized controlled trial (RCT) data by comparing rivaroxaban plus aspirin versus aspirin alone in patients with PAD, with simultaneous evaluation of efficacy and safety outcomes.

## Methodology

This systematic review and meta-analysis study was conducted according to the Preferred Reporting Items for Systematic Reviews and Meta-Analyses (PRISMA)^[^21^]^.

## Data sources and search strategy

A comprehensive literature search was conducted on PubMed, Scopus, Cochrane Library, and Google Scholar from inception until July 2025. The search strategy employed the following key terms and Boolean operators: (“Peripheral Arterial Disease”[Mesh] OR “Peripheral Artery Disease”[tiab] OR PAD[tiab] OR “Peripheral Vascular Disease”[tiab]) AND (“Rivaroxaban”[Mesh] OR rivaroxaban[tiab]) AND (“Aspirin”[Mesh] OR aspirin[tiab] OR acetylsalicylic acid[tiab])AND (“Randomized Controlled Trial”[pt] OR randomized[tiab] OR randomised[tiab]). The search was limited to studies published from database inception to July 2025, with no language restrictions. The full search syntax for each database is provided in Supplemental Digital Content Table S1, available at: http://links.lww.com/MS9/B179.

## Study selection

We included RCTs comparing rivaroxaban (any dose) plus aspirin versus aspirin alone (or placebo) in adult patients (≥18 years) with clinically diagnosed PAD. Exclusions included non-randomized studies, trials involving additional antithrombotic agents, pediatric populations, and studies with fewer than 100 total participants. Review articles, commentaries, and case reports were excluded.

## Data extraction

The retrieved articles were exported to Rayyan.ai^[^22^]^ where first, duplicate articles were removed from the list. Next, two reviewers (D.K. and M.F.) carefully evaluated the remaining articles, and only those which satisfied the aforementioned eligibility criteria were included. The first screening process was based on title and abstract. A subsequent screening process was performed on the remaining articles, based on their full texts. A third facilitator was approached to settle any conflicts regarding the results. Data for baseline characteristics and outcomes, retrieved from the completed RCTs, were used to create an online Google Spreadsheet. Baseline characteristics included mean age (SD), gender, body weight, race, baseline BMI, comorbidities, and previous MI, stroke, and eGFR. The outcomes included were:

### Efficacy outcomes

The primary outcome is the occurrence of MACE, defined as a composite of MI, ischemic stroke, and CV death. Outcomes were assessed as dichotomous variables (event vs no event) over the follow-up period.

Secondary outcomes included MI, ischemic stroke, major amputation, CV death, and ALI. These outcomes are also assessed as dichotomous variables (event vs no event) over the follow-up period.

### Safety outcomes

Safety outcomes included fatal bleeding, intracranial hemorrhage, the composite of fatal and intracranial bleeding, and thrombolysis in MI (TIMI) major bleeding. All safety outcomes were assessed as dichotomous variables (event vs no event) over the follow-up period.

## Study the risk of bias assessment

The Cochrane Risk-of-Bias 2 (RoB-2) tool was used to assess the risk of bias in all included RCTs, in accordance with PRISMA 2020 recommendations^[^23^]^. Two reviewers (D.K. and M.F.) independently assessed the risk of bias for each included study using the Cochrane Risk-of-Bias 2 (RoB-2) tool. Disagreements were resolved through discussion or by consulting a third reviewer (D.Z.). Assessments were performed at the outcome level across five domains: randomization process, deviations from intended interventions, missing outcome data, measurement of the outcome, and selection of the reported result.

## Statistical analysis and data synthesis

The meta-analysis was conducted using Review Manager (RevMan version 5.4; Copenhagen: The Nordic Cochrane Centre, The Cochrane Collaboration)^[^24^]^. Dichotomous outcomes were pooled and presented as risk ratios (RRs) with 95% confidence intervals (CIs). When at least two studies with comparable design and outcomes were available, a meta-analysis was performed using a random-effects model (DerSimonian and Laird method). Forest plots were generated to visually summarize the pooled effect estimates.

Statistical heterogeneity among studies was assessed using the *I*^2^ statistic, with values of approximately 25, 50, and 75% representing low, moderate, and high heterogeneity, respectively. An *I*^2^ value greater than 50% was considered indicative of substantial heterogeneity. Where quantitative synthesis was not feasible due to limited data or methodological heterogeneity, findings were summarized narratively.

## Publication bias

Publication bias was assessed for the primary efficacy outcome (MACE) by visual inspection of funnel plots and by Egger’s regression test, using Comprehensive Meta-Analysis software. Statistical significance was set at *P* < 0.05^[^25^]^.

## Certainty of evidence assessment

GRADE pro^[^26^]^ was used to assess the certainty of the evidence for the primary and secondary outcomes. Each outcome was evaluated for risk of bias, inconsistency, indirectness, imprecision, and potential publication bias. The final certainty ratings (high, moderate, low, or very low) are reported alongside the findings.

## Result

### Study selection

**Figure 1.**
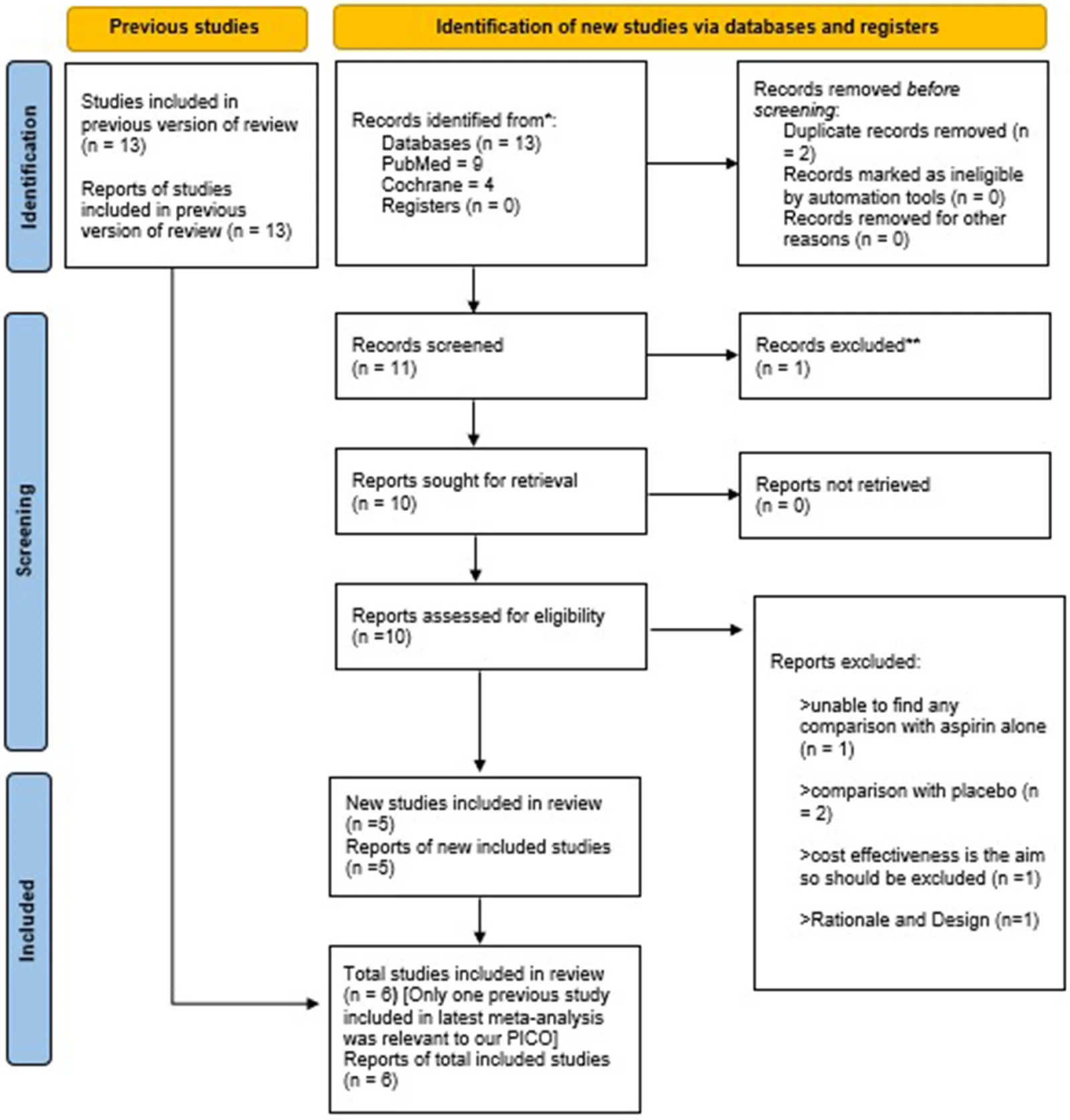
PRISMA flow chart.

The searches yielded 13 articles from PubMed and Cochrane Central databases. After filtering duplicates and excluding irrelevant articles based on title, six articles were examined for the availability of full texts and data related to the research objective. Following the assessment of complete texts, six articles were included for analysis. The study selection steps are presented in Figure 1.

### Characteristics of studies

Both male and female patients were included in these trials. These six RCTs ultimately conducted 90 325 participants to study the effects of rivaroxaban plus aspirin involving both the male and female patients of different age groups (Table 1).

**Table 1:** Baseline characteristics.

Characteristic	Yan Liang (2020)	Marc P. Bonaca (2020)	Morji Krantz (2021)	John W. Eikelboom (2020)	Eric Kaplovitch (2020)	Kelley R.H. Branch (2023)
Study design	RCT	RCT	RCT	RCT	Subanalysis of RCT	RCT
Total population	27 395	6564	6564	18 278	4129	27 395
Gender (M/F)	14 230/4048	4857/1707	4860/1704	14 257/4021	2932/1197	21 375/6020
Mean age	Women: 69 ± 8	Not mentioned	68.2 ± 7.9	66.8 ± 8.8	Not mentioned	68.2 ± 7.9
	Men: 68 ± 7.9					
Mean BMI	Women: 28.7 ± 5.6	Not mentioned	Not mentioned	28.4 ± 4.7	Not mentioned	28.3 ± 4.7
	Men: 28.2 ± 4.5					
Intervention	Rivaroxaban + Aspirin	Rivaroxaban + Aspirin	Rivaroxaban + Aspirin	Rivaroxaban + Aspirin	Rivaroxaban + Aspirin	Rivaroxaban + Aspirin
Control	Aspirin only	Placebo + Aspirin	Aspirin monotherapy	Aspirin only	Aspirin only	Aspirin only
Intervention group, N	9139	3286	3286	9152	2770	18 269
Control group, N	9126	3278	3278	9126	1359	9126
Hypertension (%)	Women: 80.4%	81.7%/81.1%	Age ≤75: 79.48%	75.1% alive	Not mentioned	No events: 75%
						1 event: 81.5%
				82.6% died		
						≥2 events: 86.8%
			Age >75: 88.87%			
	Men: 74.0%					
Diabetes mellitus (%)	Women: 41.5%	0.4	Age ≤75: 39.61%	Not mentioned	0.47	37.3%
	Men: 36.9%					
			Age >75: 41.80%			
Previous MI (%)	Women: 54.3%	0.11	10.87%/10.90%	0.622	Not mentioned	0.619
	Men: 64.5%					
Previous stroke (%)	Women: 4.0%	2.2%/2.5%	Not mentioned	0.036	Not mentioned	0.036
	Men: 3.7%					
Heart failure (%)	Women: 22.8%	Not mentioned	Not mentioned	0.135	Not mentioned	0.211
	Men: 21.2%					
eGFR < 60 (%)	Women: 32.5%	0.2	Age ≤75: 14.79%	0.269	Not mentioned	0.223
			Age >75: 41.58%			
	Men: 20.1%					
CAD prevalence (%)	Women: 83.5%	0.31	Not mentioned	Not mentioned	Not mentioned	Not mentioned
	Men: 92.7%					
PAD prevalence (%)	Women: 35.4%	Not mentioned	Not mentioned	Not mentioned	Not mentioned	0.269
	Men: 25.0%					

BMI, body mass index; CAD, coronary artery disease; eGFR, Estimated Glomerular Filtration Rate (ml/min/1.73 m^2^); M/F, male/female; MI, myocardial infarction; PAD, peripheral artery disease; RCT, randomized controlled trial.

#### Gender distribution

The included trials enrolled both male and female participants, with the proportion of women varying across studies. In trials focusing specifically on PAD populations, women represented approximately 40–45% of participants. The variation reflects differences in study design and eligibility criteria. For example, the COMPASS subgroup analysis by Liang *et al*, which included patients with either coronary or PAD, reported 4048 women (22.1%) and 14 230 men (77.9%) within its comparison of combination therapy vs aspirin. In contrast, the study by Branch *et al*, which had different inclusion criteria, reported a higher proportion of women.

#### Age groups

Reported mean ages of participants ranged from approximately 66.8 to 69.0 years, indicating that the study population was predominantly elderly and at high risk for CV events.

#### Comorbidities considered

Comorbidities were recorded as multiple among the different studies. CV and metabolic diseases are as follows: hypertension was the most frequently reported condition, with prevalence ranging from 81.0 to 86.8% in those studies that reported it; diabetes mellitus comorbidity recorded in between 36.9 and 41.5% of participants, again depending on the study. Previous MI was noted in quite a few studies; the prevalence went as high as 54.3% in the study by Yang Liang .Stroke was less frequently mentioned but ranged from 2.4 to 3.6% where reported. Heart failure and chronic kidney disease (eGFR < 60 mL/min/1.73 m^2^) were included in some of the studies. For example, Branch *et al* has reported eGFR <60 in 22.3% of patients. PAD has been discussed in a few studies. In the work of Yang Liang, the prevalence is 35.4% among women and 25.0% among men.

### Risk of bias assessment

The risk of bias for each included study was evaluated using the Cochrane Risk of Bias Tool 2 (RoB 2) applicable to five key domains (D1–D5) across RCTs. The domains are rated on how likely it is that bias has been introduced into the study results.

#### Domains are as follows

D1 – Randomisation process, D2 – Deviations from intended interventions, D3 – Missing outcome data, D4 – Measurement of the outcome, & D5 – Selection of the reported result.

#### Color coding

Green = Low risk of bias, Yellow = Some concerns, & Red = High risk of bias.

#### Overview result

In the six studies, three of them (Bonaca 2021, Kaplovitch 2020, and Branch 2023) have recorded an overall low risk of bias. One study (Branch 2023) has some concerns-yellow. Two studies (Eikelboom 2021 and Yan Liang 2020) were assessed as having an overall high risk of bias. That places it at: one study, about 16.7%, had a few concerns. The results of the risk of bias assessment and summary are presented as Supplemental Digital Content Figure1, available at: http://links.lww.com/MS9/B179 and Supplemental Digital Content Figure 2, available at: http://links.lww.com/MS9/B179.

### Efficacy evaluation

Impact of rivaroxaban plus aspirin vs aspirin alone on PAD.

### Primary outcome

#### MACE

**Figure 2.**
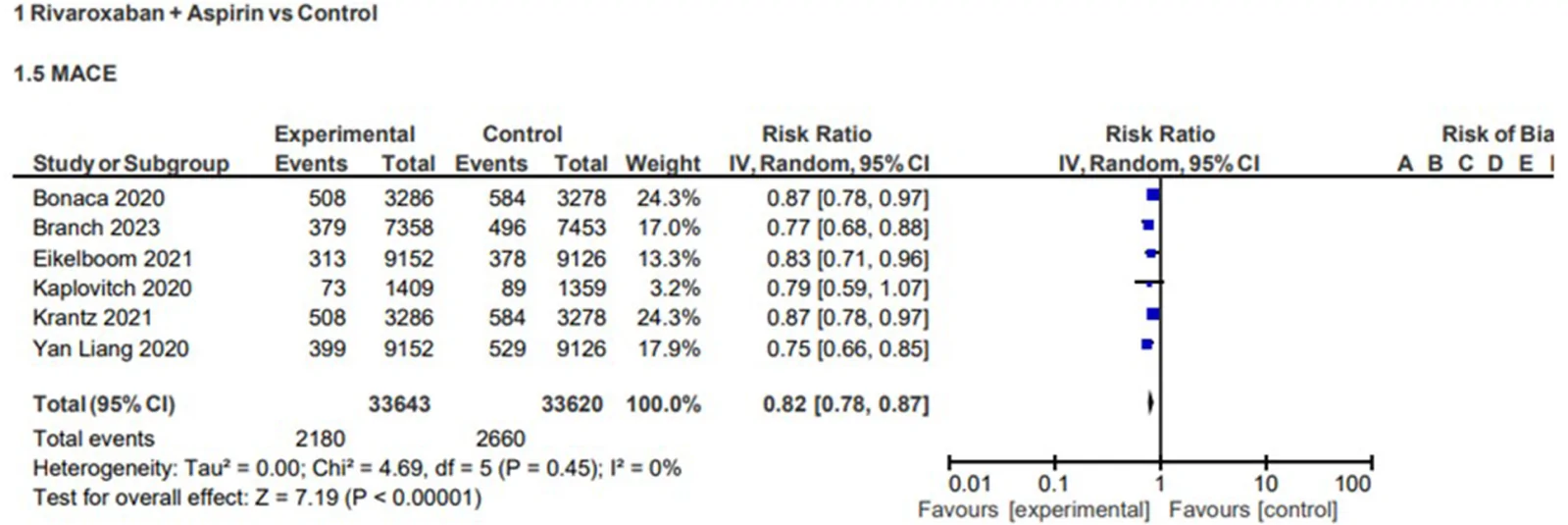
Forest plot of MACE.

Figure 2 shows a forest plot in terms of MACE. A statistically significant reduction in MACE was found in pooled six trials when rivaroxaban plus aspirin was compared to control. The risk ratio came to 0.82, and the 95% confidence interval ranged between 0.78 and 0.87. The intervention reduced the relative risk by 18%, which is a clinically meaningful improvement in patients who are at high risk for developing major atherothrombotic events. Statistical confidence: with such a narrow CI and an extremely significant *P*-value, it is highly unlikely that this effect has occurred due to mere chance. No statistical heterogeneity was observed (*I*^2^ = 0%, Chi^2^ *P* = 0.45), meaning that there are no differences between the included trials and results can be relied upon as well generalized regarding effect. Finding helps with the use of rivaroxaban plus aspirin as a viable strategy for vascular protection in high-risk individuals.

### Secondary outcomes

#### Myocardial infarction

**Figure 3.**
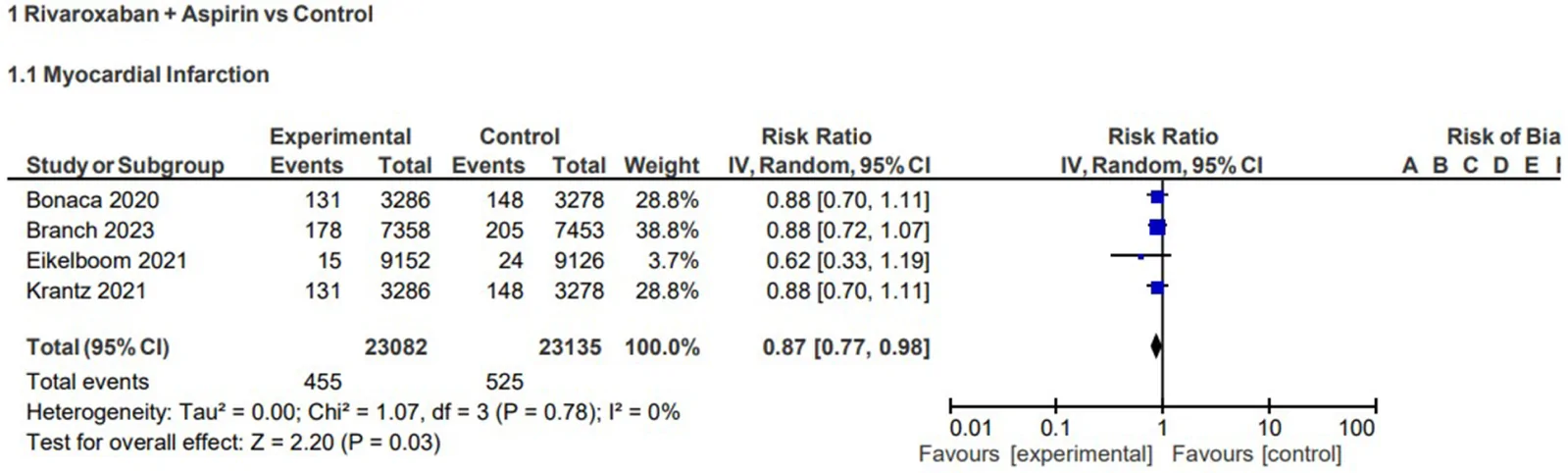
Forest plot of myocardial infarction.

Figure 3 shows a forest plot in terms of MI. Results from four trials reported a MI RR of 0.87 (95% CI: 0.77–0.98), *P* = 0.03 in favor of the experimental group. Meaning: it translates into a 13% reduced risk of getting an MI by using combination therapy. Inconsistency among studies: *I*^2^ = 0%. Although prevention of MI is clinically very valuable especially in patients with prior events or established CAD, this modest relative reduction makes it less emphasized. These results further solidify the concept that dual pathway inhibition would be the axes around which improvement of both plaque stabilization and thrombotic prevention pivot.

#### Ischemic stroke

**Figure 4.**
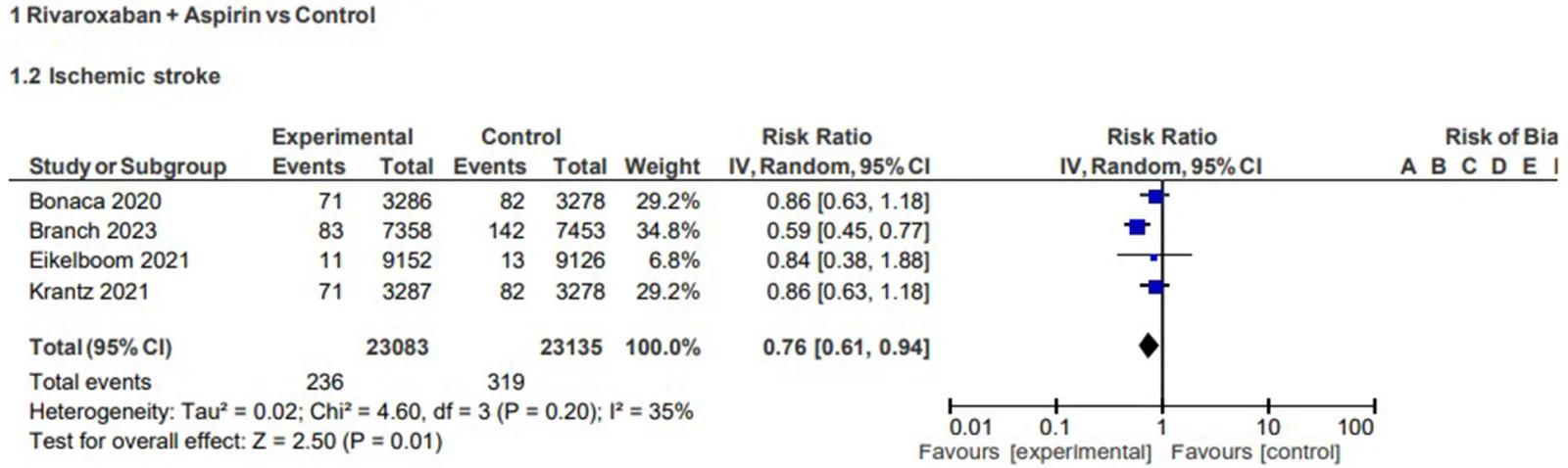
Forest plot of ischemic stroke.

Figure 4 shows a forest plot in terms of ischemic stroke. Risk for ischemic stroke was significantly lower in the intervention group (RR: 0.76, 95% CI: 0.61–0.94, *P* = 0.01) based on four studies. A relative risk reduction of 24% for an outcome that can be so devastating. *I*^2^ = 35% (Chi^2^ *P* = 0.20) meaning moderate heterogeneity. Variability does not rise to the level of being statistically significant. This reiterates the advantage of the combination in cerebrovascular protection, which may add value in patients with polyvascular disease or prior stroke.

#### Cardiovascular death

**Figure 5.**
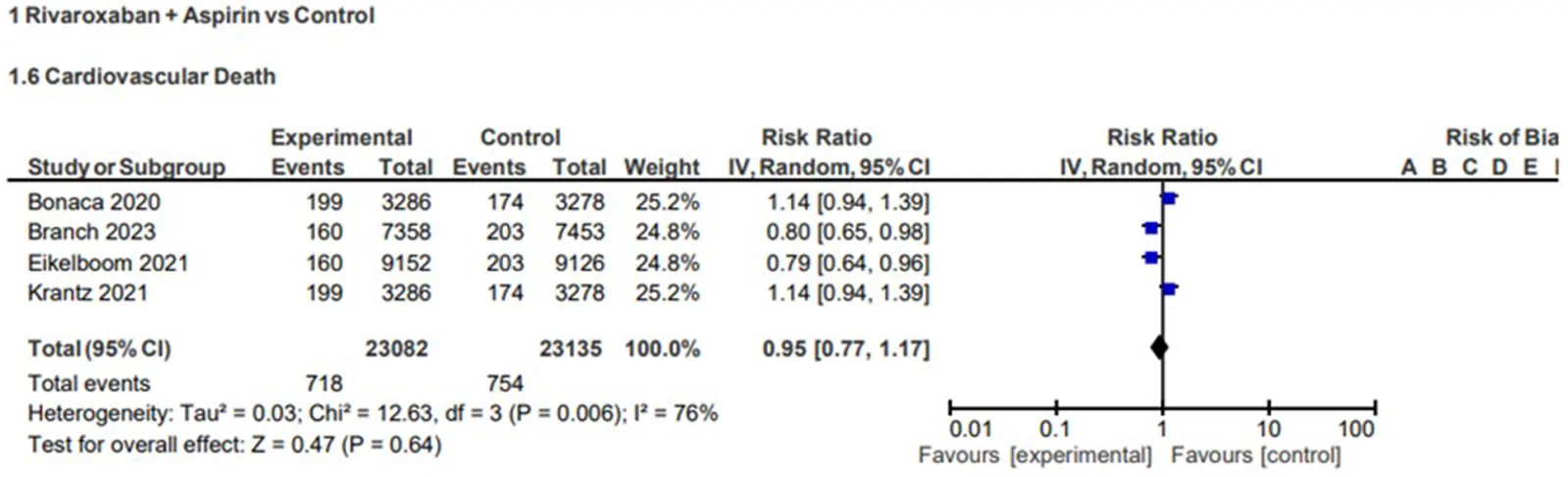
Forest plot of cardiovascular death.

Figure 5 shows a forest plot in terms of CV mortality. There was no statistically significant difference in CV mortality (RR: 0.95, 95% CI: 0.77–1.17, *P* = 0.64). Heterogeneity is high (*I*^2^ = 76%, Chi^2^ *P* = 0.006) and reduces confidence in pooled estimates. Variability may come from population risk profiles, definitions of CV death, and length of follow-up. Even though the direction of effect favors rivaroxaban plus aspirin, inconsistency across studies makes this result uninterpretable because it is not statistically significant.

#### Major amputation

The pooled risk ratio for major amputation is 0.81 (95% CI: 0.64–1.04) *P* = 0.09 is not significant. Heterogeneity is moderate (*I*^2^ = 45%) with meaningful variability between studies. As it does not reach statistical significance, the trend toward reduced amputations is in line with the reduced risk of limb ischemia. Low event rates or even underpowered analysis may have contributed to the lack of significance. In summary, results are suggestive and have no finality. This benefit needs to be confirmed, requiring more trials or longer follow-up. The forest plot of Major amputation is presented in Supplemental Digital Content Figure 3, available at: http://links.lww.com/MS9/B179.

#### Acute limb ischemia

The combination therapy substantially reduced the incidence of ALI (RR: 0.68, 95% CI: 0.59–0.78, *P* < 0.00001). A 32% risk reduction was observed, the largest among all primary outcomes, with *I*^2^ = 0%, demonstrating excellent consistency across studies. This advantage underscores the potency of rivaroxaban in forestalling thrombotic complications that extend beyond the coronary and cerebral regions. The forest plot of Acute limb ischemia is presented in Supplemental Digital Content Figure 4, available at: http://links.lww.com/MS9/B179.

## Safety evaluation

### Fatal bleeding

Though a higher trend was noted (RR: 1.38, 95% CI: 0.93–2.05, *P* = 0.11), statistical significance was not attained. The wide confidence interval crossing 1 does make it seem as though this could be a chance finding. No heterogeneity was observed (*I*^2^ = 0%), so results are consistent. Though not final, the upper CI shows a possible clinically meaningful risk that doctors have to keep in mind when managing patients with high bleeding risk.

The forest plot of fatal bleeding is presented in Supplemental Digital Content Figure 5, available at: http://links.lww.com/MS9/B179.

### Intracranial hemorrhage

No change was seen between groups (RR: 1.00, 95% CI: 0.74–1.36, *P* = 0.99) meaning that the mix does not raise brain bleeding risk. No difference was found among studies (*I*^2^ = 0%). This is comforting considering the devastating results of intracranial hemorrhage. It implies that even though the general risk of bleeding goes up, the most dreaded complication does not become substantially more probable with combination therapy. The forest plot of intracranial is presented in Supplemental Digital Content Figure 6, available at: http://links.lww.com/MS9/B179.

### Fatal and intracranial bleeding

Combined fatal bleeding or intracranial hemorrhage shows (RR: 0.89, 95% CI: 0.56 to −1.42, *P* = 0.63, *I*^2^ = 0%). There was no statistical difference between the groups. Though the point estimate favors the intervention slightly, with such a wide CI and no significance, there is no net benefit or harm that can be clearly decided upon. The result was consistent across studies. The forest plot of fatal and intracranial bleeding is presented in Supplemental Digital Content Figure 7, available at: http://links.lww.com/MS9/B179.

### TIMI major bleeding

There was a statistically significant increase in major bleeding under TIMI with rivaroxaban plus aspirin (RR: 1.41, 95% CI: 1.07–1.84, *P* = 0.01). No heterogeneity was observed (*I*^2^ = 0%), showing a consistent increase in risk across studies. This means a 41% relative increase in serious bleeding events. This is the main trade-off for the CV benefit. Although it works well in terms of event prevention, it raises major bleeding risk as well, thus making patient selection an important factor. The forest plot of TIMI major bleeding is presented as Supplemental Digital Content Figure 8, available at: http://links.lww.com/MS9/B179.

### Publication bias

Egger’s regression test indicated potential small-study effects, with an intercept of 30.39 and a statistically significant two-tailed *P*-value of 0.021, suggesting the possible presence of publication bias. Visual inspection of the funnel plot further supported this finding, as the distribution of studies appeared asymmetric around the pooled effect estimate (Supplemental Digital Content Figure 9, available at: http://links.lww.com/MS9/B179). Rather than being evenly scattered on both sides of the summary effect, a greater number of studies were concentrated on one side of the funnel, which may indicate the absence or underrepresentation of smaller studies with less favorable or non-significant results. In addition, the wide dispersion of the remaining studies suggests potential heterogeneity, implying that variations in methodology or participant characteristics may also contribute to the observed asymmetry (Supplemental Digital Content Figure 9, available at: http://links.lww.com/MS9/B179).

### Certainty of evidence

We assessed the quality of the evidence of rivaroxaban plus aspirin versus aspirin alone in patients with PAD with that expressed by the GRADE approach. For most of the major outcomes such as ALI, MI, ischemic strokes, and major CV events, the evidence was strongly in favor. Therefore, these events were rated as high certainty, i.e., one can trust the results to a great extent. However, for major amputations, the evidence profile differed. There was not much clustering, and outcomes varied widely. Therefore, we assigned it a moderate certainty grade because of serious imprecision. The evidence for CV mortality was less clear. Studies did not all agree with each other, and the numbers were not that precise either. Thus, this outcome was rated low certainty because of inconsistency and imprecision. Regarding fatal bleeding and intracranial bleeding, we rated both as moderate certainty. This was due to the fact that the number of events was small, which tended to make the results less exact. On the whole, it appears that the augmentation of aspirin with rivaroxaban helps to decrease vascular and limb complications in patients with PAD. However, this is at the expense of an increased risk of major bleeding events; and thus, the pros and cons have to be weighed by the physician. Most of the evidence is robust, however there remain a few areas that require better data for confirmation. The GRADE assessment table and its explanation is presented in Supplemental Digital Content Table 1, available at: http://links.lww.com/MS9/B179.

### GRADE working group grades of evidence

#### High certainty

We are very confident that the true effect lies close to that of the estimate of the effect.

#### Moderate certainty

We are moderately confident in the effect estimate; the true effect is likely to be close to the estimate of the effect, but there is a possibility that it is substantially different.

#### Low certainty

Our confidence in the effect estimate is limited; the true effect may be substantially different from the estimate of the effect.

#### Very low certainty

We have very little confidence in the effect estimate; the true effect is likely to be substantially different from the estimate of effect.

## Discussion

PAD is a significant worldwide health concern due to its association with poor CV outcomes and limb related complications^[^27^]^. Antiplatelet medication is the therapeutic cornerstone in the management of PAD. However, the risk of atherothrombotic events continues to exist despite conventional care, leading to the need of personalized treatment methods^[^28^]^. Rivaroxaban, which is a factor Xa inhibitor, when used alongside aspirin provides a dual path inhibition approach which may enhance the protection against arterial thrombotic events^[^29–31^]^. The primary goal of this meta-analysis was to investigate the efficacy and safety of rivaroxaban plus aspirin compared to aspirin alone in PAD patients. The study was conducted by examining six RCTs including 90 325 individuals, focusing on MACE, limb-related outcomes, and bleeding risks.

The efficacy outcomes demonstrated that the combination therapy offers significant advantages. Rivaroxaban plus aspirin was shown to have a considerably lower risk of MACE (RR: 0.82, 95% CI: 0.78–0.87), MI (RR: 0.87), ischemic stroke (RR: 0.76), and ALI (RR: 0.68), all with low heterogeneity (*I*^2^ = 0–35%) and were rated as having a high certainty of evidence^[^32^]^. These findings were consistent with those obtained from the COMPASS and VOYAGER-PAD trials, both of which revealed that dual therapy would provide significant protection to the CV system^[^33–35^]^. In patients with PAD, the decrease in ischemic stroke and ALI is noteworthy, as it highlights the advantages of rivaroxaban for the peripheral and cerebrovascular systems^[^36^]^. However, there was no significant reduction in the death rate associated with CV disease (RR: 0.95, *P* = 0.64). Furthermore, the high heterogeneity (*I*^2^ = 76%) showed inconsistent results, which led to a low certainty of evidence. In addition, the major amputation (RR: 0.81, *P* = 0.09) failed to reach statistical significance and was rated as moderate certainty due to the imprecision. These findings correspond with a trial by Bonaca *et al*, which reported that in patients with PAD, rivaroxaban plus aspirin reduced the risk of major amputation, ALI, and CV events when compared to aspirin alone^[^36^]^.

Egger’s regression test indicated a statistically significant intercept (*P* = 0.021), suggesting possible publication bias, perhaps resulting from the non-publication of minor or negative trials. This highlights the need of cautious interpretation, as bias may lead to an overestimation of the effects of the intervention^[^37^]^.

Among the secondary outcomes that primarily focused on safety, a significant increase was observed in TIMI major bleeding (RR: 1.41, *P* = 0.01). The findings were consistent across trials (*I*^2^ = 0%) and were graded as high certainty. This finding is in line with a study by Gibson *et al*, conducted in stabilized patients with post-acute coronary syndrome which reported that when compared to aspirin alone. Rivaroxaban plus aspirin exhibited a significant increase in TIMI major bleeding^[^38^]^. There was no significant difference in fatal bleeding (RR: 1.38, *P* = 0.11) and intracranial bleeding (RR: 1.00), but the confidence intervals were large and the certainty was modest due to very few event counts. In addition, there was no significant difference between the combined fatal and intracranial bleeding (RR = 0.89; *P* = 0.63). The findings of this research study are consistent with those of a trial that found that the incidence of combined fatal and intracranial bleeding did not vary significantly between patients with PAD who were given rivaroxaban plus aspirin and those who were given aspirin alone^[^36^]^. These findings align with prior studies such as Debasu *et al* and Anand *et al* that illustrated that rivaroxaban plus aspirin is more effective than aspirin alone in reducing CV events; however, it does raise the risk of severe bleeding but not the intracranial or fatal bleeding^[^33,39^]^. In clinical decision-making, the balance of efficacy and harm continues to be of extreme significance^[^40^]^.

Clinical decision-making in PAD requires individualized assessment of ischemic and bleeding risk. Patients with high ischemic burden such as those with polyvascular disease, prior revascularization, diabetes, or diffuse atherosclerosis may derive greater net benefit from dual pathway inhibition with rivaroxaban plus aspirin. Conversely, patients with prior major bleeding, advanced renal dysfunction, anemia, or frailty may be better suited for single antiplatelet therapy. Treatment selection should therefore be tailored to patient-specific risk profiles rather than applied uniformly.

Rivaroxaban with aspirin has been reported in a study to minimize major vascular events in PAD patients at high bleeding risk, therefore future research should focus on these individuals^[^40^]^ or individuals not included in the current studies, as women with PAD, elderly people, and people with chronic kidney diseases, who would have distinct risk-benefit profiles of rivaroxaban with aspirin^[^32^]^. To find the long-term balance between efficacy and harm, longer follow-up trials are required. In order to maximize dual options for therapy, studies ought to inquire into specific risk stratification tools. Lastly, the design of prediction models that integrate biomarker and clinical data could be helpful in the identification of patients who benefit the most from combination therapy^[^41^]^.

### Strengths

This study has various strengths. It includes a large sample size comprising over 90 325 participants from six high-quality RCTs, which improved the generalizability of the results across diverse populations. The use of robust approaches such as GRADEpro for evidence certainty and Egger’s regression for bias strengthens the reliability of conclusions^[^42^]^. The primary outcomes show low heterogeneity, which suggests consistency across populations and settings^[^43^]^. In addition, the review followed PRISMA guidelines and used the Cochrane RoB 2 tool for a comprehensive risk-of-bias assessment, which enhances the methodological transparency.

### Limitations

There are some limitations of this study. Firstly, the CV death outcome shows high heterogeneity, which limits confidence in pooled results. Secondly, moderate to high risk of bias in two included trials (Eikelboom 2021 and Yan Liang 2020) may compromise internal validity. Publication bias is a concern, as identified via Egger’s regression. Moreover, no patient-level data were available, limiting subgroup analysis by comorbidities or demographic characteristics. The limited event rates of major amputation and bleeding outcomes in PAD lowered statistical power and precision^[^44^]^. Lastly, differences in intervention duration and follow-up time between trials may influence the pooled findings^[^45^]^.

## Conclusion

This meta-analysis highlights that rivaroxaban plus aspirin significantly reduces MACE, MI, ischemic stroke, and ALI in PAD patients, notwithstanding an elevated risk of major bleeding. Although the primary outcomes and most of the secondary outcomes are of high certainty of evidence, bleeding risks and several secondary outcomes require cautious interpretation. Individual bleeding risk should be accounted for when applying dual therapy in clinical settings, and future trials are required to clarify patient selection and evaluate long-term outcomes.

## Data Availability

Data generated are present in manuscript and its supplementary files.

## Research registration unique identifying number (UIN)

1. Name of the registry: PROSPERO 2. Unique Identifying number or registration ID: CRD420251087922 3. Hyperlink to your specific registration (must be publicly accessible and will be checked): https://www.crd.york.ac.uk/PROSPERO/view/CRD420251087922.
